# Medical Opinion and Movement

**Published:** 1910-09-10

**Authors:** 


### Medical Opinion and Movement.

### Scarlet Red for Epithelial Growth.

Fisher first observed some few years back that solutions of scarlet red subcutaneously injected into a rabbit's ear induced an active proliferation of the overlying epithelium. As a result of this observation scarlet red has been employed to assist in the skin formation over clean granulating surfaces, and has been found of great value for this purpose, hastening the healing of large cutaneous defects and not infrequently supplanting skin grafting. Dr. Strauss publishes an article on the subject in the *Deutsche Medizinische Wochenschrift*. He employs an 8-per-cent. ointment. Scarlet red is dissolved in chloroform oil, the solution being stirred until the chloroform has evaporated. Vaseline is then added to make the 8-per-cent. ointment. This is spread upon gauze and is applied to the cleansed area. It should be changed every day or at least every other day. The addition of any antiseptic is unnecessary. This treatment is indicated in any large granulating area and is of special value for the epidermisation of defects following burns and for granulating wounds. In such cases, according to this author, subsequent contracting scars are in a large measure avoided. It has also acted very well in cases of ulcers of the leg, especially of the varicose variety and in weeping eczema.

### Cerebrospinal Fluid and Visual Trouble.

An interesting case is recorded by Dr. E. Velter in the *Annale d'oculist* of amblyopia accompanied by other nervous symptoms due to a hypertension of the cerebrospinal fluid and relieved by lumbar puncture. The patient was a man aged forty-two, alcoholic, who was suddenly attacked on two different occasions within a few months of each other with severe pain in the head, noises in the ears, nystagmus, stupor, and a dulling of the intelligence —all symptoms of increased intracranial pressure. There were at the same time signs of a mild toxic polyneuritis of the pseudo-tabes type and visual troubles suggestive of a papillary stasis, but with no ophthalmoscopic changes, except perhaps a slight paleness of the temporal half of both papillæ. The visual troubles consisted in a moderate amblyopia with a slight retraction of the peripheral visual field, but without any central scotoma. Although visual acuity appeared sufficient for proper vision, nevertheless the patient was seriously inconvenienced by his condition. He could not pursue his work as coachman, could not recognise objects and people around him, and could hardly direct himself. The author suggests that this extreme disability out of proportion to his actual visual deficiency may have been due to trouble set up in the central paths of visual perception, either by compression from intracranial hypertension or by central œdema. In any case, lumbar puncture, allowing the extraction of a few cubic centimetres of cerebrospinal fluid under high pressure rapidly and completely relieved the condition. Examination of this liquid showed nothing abnormal and not a trace of meningeal reaction.

### Bier's Passive Hyperæmia.

It may be remembered that Bier's method of hyperæmic congestion has been used successfully in the treatment of writer's cramp, cases being recorded by Dr. Bucciante and by Dr. Hartenberg. Dr. Migliaccio has now published the report of a case of alcoholic tremors which he was able to completely relieve by the same method of treatment. The patient was a workman aged fifty, who had been addicted to alcoholic excess for a long while, and was suffering from severe gastric dyspepsia. One day in the course of his work he was taken with an acute pain at the level of the left radiocarpal joint (the patient was left-handed). Under appropriate treatment this pain disappeared in the course of a month, but left a tremor of the hand, which increased on voluntary movement. The whole condition—the tremor and the gastritis were undoubtedly of alcoholic origin. Special diet and total abstention from alcohol were prescribed, and at the same time the tremors of the hand were treated by Bier's method. Already after six applications, each of which was of about 1½ hours' duration, there was marked improvement, which continued to increase after each successive séance, so that at the end of a month's treatment the patient was completely cured of these tremors. The rapidity of the improvement and the progress taking place *pari passu* with the applications proved beyond doubt, at any rate to the mind of the author, that the cure was actually due to the passive hyperæmia and not simply to the special diet and abstention from alcohol.

### Intra-uterine Poliomyelitis.

In a suggestive paper contributed to *Brain* Dr. Batten propounds the query, Does poliomyelitis occur during intra-uterine life? and proceeds to examine certain evidence which leads him to the belief that such an event is possible. Flaccid paralysis of one arm, due to birth-injury, is, as the author remarks, quite common; but the possibility that in a small proportion of such paralyses the real lesion may be in the anterior horn cells, not in the brachial plexus, seems to have occurred also to Fritsch, who published the case of a child born without injury (but breech first) paralysed in both arms and both legs. In Fritsch's case no pathological examination was made, so the case remains doubtful. From time to time Dr. Batten has seen infants paralysed at, or immediately after, birth, in whom a diagnosis of intra-uterine poliomyelitis has seemed plausible; but he has seen only two cases in which this presumption has been abutted by post-mortem examination. These he narrates in detail: in the first the chain of evidence is not complete, but is certainly very strong; in the second there is no doubt about the lesion, but some uncertainty whether or not it existed at birth. The author summarises that there is good, but not conclusive, evidence in favour of the assumption that poliomyelitis does occur during intra-uterine life.

### Hydramnios.

Dr. Burstal records briefly in the *Practitioner* the statistics of the York Road Lying-in Hospital from 1894 to 1904, and of Queen Charlotte's Hospital from 1900 to 1905, in respect of hydramnios. He takes three pints as the limit of the normal amniotic fluid, and treats of everything in excess of this as constituting hydramnios. Altogether he found recorded 133 cases of this condition, being 1.3 per cent. of cases at York Road and 0.65 per cent. of those at Queen Charlotte's. There were twins in six of the cases, a fact of which there seems no obvious explanation; twins, together with hydramnios, must distend the uterus to a most remarkable degree. Seventy-four of the children were males, 61 females; this preponderance of males is contrary to the usually current statements of best books. Four of the children were deformed, including two which were anencephalic. No marked influence on the second stage of labour came to light; the average duration was one and a-half hours. Nor did abnormal presentations occur more often than usual, considering the six twin pregnancies. The ages of the mothers varied from eighteen to forty-six, and the pregnancy concerned varied from the first to the fifteenth; in not a single case was there any history of a previous hydramnios. Albumin was found in 46 out of 112 recorded cases. Hydramnios, therefore, must still be regarded as one of those mysterious events whose cause is still entirely conjectural.

### Vincent's Angina.

In the *British Journal of Children's Diseases* Dr. J. D. Rolleston publishes his experience of thirty-two cases of Vincent's angina, which is defined as a faucial lesion, usually of unilateral distribution, characterised by deep ulceration of the tonsil and adjacent structures, a peculiar fœtor and enlargement of the corresponding lymph glands. Two organisms are usually found ætiologically associated with this disease; one a spirillum and the other a fusiform bacillus. The ulcerative lesion is commonly the later stage of a membranous initial one, and the resemblance of the latter to diphtheria clinically may be very great; the most absolutely characteristic sign is the fœtor, which is peculiar to itself. Vincent's angina is not common; it is found in 0.9 per cent. of all cases of sore throat taken to the Metropolitan Asylums Board Hospitals; and in 4.9 per cent. of cases of non-diphtheritic angina. It is practically confined to children between two and eighteen; it is very little, if at all, contagious, and is most often met with in the spring. It is not commoner in weakly children or in those with oral sepsis; and it is not often associated with other diseases. The constitutional symptoms are slight or absent, whereas the local affection is more pronounced than in diphtheria. The prognosis is favourable; complications are infrequent and usually insignificant. Treatment consists in the local application of iodine or methylene-blue powder: internal medication is usually unnecessary.

### Skin Rashes in Typhoid Fever.

Excluding altogether the common rose rash of typhoid fever, there are other skin eruptions occasionally met with during the course of that disease. Dr. J. Phillips collects in the *American Journal of the Medical Sciences* all those observed in 1,230 cases of enteric treated at the Lakeside Hospital. Herpes was present twelve times; eight times as labial herpes during the first week (which was at one time held to exclude typhoid from the diagnosis), and four times later in the disease upon the body. Urticaria was noted twenty-one times; no quinine was given to any of the cases. Sudamina are not very uncommon, and Dr. Phillips supports the opinion of De Lacaze that a crop of these vesicles in the third or fourth week is a good prognostic indication. Erythema developed in seven patients. Desquamation took place in eighty-three patients, following either rose rash, sudamina, or erythema; or as a trophic change analogous to the falling-out of the hair. Measly and scarlatiniform rashes have been recorded occasionally, but there were none in this series. Purpura was noted in six cases, and a pemphigoid eruption in four. Erysipelas complicated the later stages of the fever twice; both patients recovered. There were forty-five cases of furunculosis, two of carbuncle, five of onychia, nine of subcutaneous abscess, one of impetigo, and one of infected sebaceous cyst. Striæ patellares were found in a boy of fourteen, who became very emaciated in a prolonged attack, and also in a middle-aged woman.

### Apparent Cure of Pernicious Anæmia.

The fallacy of regarding any remission of symptoms, however prolonged, as a definite cure when once pernicious anæmia has been diagnosed with certainty is well shown by the record of a remarkable case published in the *American Journal of the Medical Sciences* by Professor A. McPhedran. In 1889 a physician, then aged forty-nine, had to give up work owing to anæmia and weakness, which had been coming on for a year or two. He soon became very ill, with delirium and a temperature of 104° F. There were vomiting, diarrhœa, lemon-coloured skin, and retinal hæmorrhages in both eyes. The blood showed the changes characteristic of pernicious anæmia; there were 730,000 red corpuscles per cubic millimetre. He was treated with large doses of arsenic, began to improve after a month's severe illness, and in five months was apparently well. He went back to work, and continued to do it without any difficulty until 1906, when his strength began to fail; so complete was his recovery that the case was included in published statistics as a definite cure, and the patient himself never had his blood examined, because he felt so well. In 1907 he had to give up work and undergo treatment, after which he improved a good deal. He went to the Rocky Mountains for a holiday, but had a relapse there, and had to take a rest once more. In 1908 he failed steadily, and died in the summer of typical pernicious anæmia, at the age of sixty-eight. The author has found no record in the literature of any remission as long as this one of seventeen years. He remarks that had the patient died of some intercurrent illness a year or two before his relapse his case would, with good reason, have been regarded as one of permanent recovery. As a further item of interest in this particular case it is worth adding that the patient had had all his teeth removed before the original attack, and always kept his gums in perfect condition.

### Cast of Male Urethra.

Dr. A. L. Wolbarst reported to the Eastern Medical Society of America the case of a man, aged twenty-four, who, in 1908, after accidental cauterisation with strong silver solution, passed a cast of his entire urethra. Being under treatment for anterior urethritis, as the result of the transposition of two bottles, the anterior urethra was injected with 10 per cent. silver nitrate. On the mistake being discovered, the bladder was thoroughly irrigated with warm salt solution, and the patient then given some of it to use at home. There was no evidence of trouble for four days, but on the fifth the urine was cloudy, and there were signs of inflammation. There was no pain of any kind. Treatment was then instituted, which consisted in copiously irrigating the entire urethra twice daily with salt solution. Pain commenced to be present at the neck of the bladder, the temperature rose slightly, and the urine commenced to show signs of blood at the end of micturition. Broken-down leucocytes were found in the urine, but no micro-organisms. After a time there was more or less constant tenesmus and urinary urgency, for which the patient was admitted to hospital. His temperature on admission was 102.3, pulse 88, respiration 24, specific gravity of urine 1022, no albumin, and the epithelial and pus cells were negative as to gonococci. The thighs and scrotum showed superficial burns from the caustic. There was no polyuria, but the patient had vomited the night before admission. The glans penis and inner surface of the prepuce were red. A slight muco-purulent discharge oozed from the urethra. No gonococci were found in this discharge. The patient was treated by rest in bed, urotropine by the mouth, and urethral irrigations with warm boric lotion. During the next week he improved gradually, and except for the purulent discharge was normal. Three weeks after the initial accidental caustic injection, a dark tenacious mass appeared at the meatus externus, which was removed, without pain, with dressing forceps. Three days later a second mass came away. With the object of preventing the raw urethral surfaces coming together, medicated bougies were used for the next five weeks, after which a sound was passed. At first only a French 21 would pass, but gradual dilatation ended in the passing of a 28. At a later date two contractions of the urethra occurred which necessitated treatment by passing bougies once a month. The urine is now quite clear, and the gonorrhœa cured. Examination of the cast showed necrotic tissue, with areas suggestive of capillaries. In spite of the extensive destruction of tissue, new mucous membrane would seem to have been formed.

### Prophylaxis of Acute Anterior Poliomyelitis.

Regarding the prophylaxis of such a disease as anterior poliomyelitis, Römer and Joseph in the *Münchener Med. Woch.* state that two forms of preventive treatment—the hygienic and the medicinal—must be taken into account. In hygienic prophylaxis the possibility of domestic animals, such as dogs and chickens, acting as carriers must be remembered, as well as the part played by human beings in distributing the disease. The mode of transmission probably has many points in common with that of meningitis. According to some authorities, the specific organism, or poison, is to be found in the naso-pharyngeal secretions, both of those suffering from the disease and of the apparently healthy, infection being caused by contact with these secretions. As the result of experiment, it has been shown that the virus remains active after drying for a month, so that dust may play a part in the dissemination of the disease. The authors have proved by experiment that exposure to the vapour of formaldehyde for seven and a half hours, as in the ordinary method of disinfecting a room, renders the virus inert when injected into a monkey. Therefore the room of a patient who has recovered from an attack should be disinfected in this way. The same precautions with regard to the nose and mouth of the patient and all coming in contact with him are to be observed, as are the rule when dealing with other infectious diseases, such as diphtheria, etc. With regard to the medicinal part of the prophylactic treatment, much hope is placed in the use of the serum of an immunised animal. Since one attack renders an animal immune to a second, and the serum of an immunised animal has been shown to prevent the development of an attack in an inoculated one, there would seem to be grounds for believing that this hope will be realised.

### The Storage of Vaccine Lymph.

From the experiments and researches of M. Kelsch, recently reported to the Paris Academy of Medicine, it would seem to follow that vaccine preserved in the refrigerators of the city of Paris at a temperature of $-10°$ C. and $-15°$ C. not only retains its physiological action, but even acquires increased activity. On the other hand, similar vaccine kept in the ice-chests of the Academy at a temperature of 10° C. loses its activity. Thus vaccine can be kept indefinitely in a refrigerator, provided that it is examined and tested each year. The author thinks that this activity is due to the fact that the vaccine is preserved pure and without admixture of glycerine.

